# Sacral insufficiency fractures after high-dose carbon-ion based radiotherapy of sacral chordomas

**DOI:** 10.1186/s13014-018-1095-x

**Published:** 2018-08-23

**Authors:** Tilman Bostel, Nils Henrik Nicolay, Thomas Welzel, Thomas Bruckner, Matthias Mattke, Sati Akbaba, Tanja Sprave, Jürgen Debus, Matthias Uhl

**Affiliations:** 1 Department of Radiation Oncology, University Hospital Heidelberg, Im Neuenheimer Feld 400, 69120 Heidelberg, Germany; 2 Clinical Cooperation Unit Radiation Oncology, German Cancer Research Center (DKFZ), Im Neuenheimer Feld, 280 Heidelberg, Germany; 3 Heidelberg Institute for Radiation Oncology (HIRO), National Center for Research in Radiation Oncology, Im Neuenheimer Feld, 280 Heidelberg, Germany; 4 Department of Medical Biometry, University Hospital Heidelberg, Heidelberg, Germany

## Abstract

**Background:**

This study aimed to analyse the frequency and clinical relevance of sacral insufficiency fractures (SIFs) after high-dose carbon-ion based irradiation of sacral chordomas.

**Methods:**

A total of 56 patients were included in this retrospective study. Twenty one patients (37%) were treated with definitive radiotherapy (RT), and 35 patients (63%) received postoperative RT using carbon ions, either in combination with photons or as single-modality treatment (median radiation dose 66 Gy RBE, range 60–74 Gy). Follow-up examinations including MRI of the pelvis were performed at 3-monthly intervals in the first year and consecutively at 6-monthly intervals. Median follow-up was 35.5 months (range 2–83).

**Results:**

SIFs were diagnosed in 29 patients (52%) after a median follow-up of 11 months (range 1–62 months). Most sacral fractures (79%) occurred within 2 years after RT. For the overall study population, the fracture-free survival probability amounted to values of 0.68 (95% CI, 0.53–0.79) after 1 year, 0.46 (95% CI, 0.31–0.60) after 2 years, and 0.31 (95% CI, 0.16–0.47) after 5 years. Statistical analysis showed no significant difference regarding the fracture rates between patients who received an operation and postoperative RT and patients treated with definitive RT. About one third of the patients with SIFs (34%; 10 of 29 patients) had associated clinical symptoms, most notably pain. All patients with symptomatic fractures required strong analgesics and often intensive pain management.

**Conclusions:**

Sacral fractures after high-dose carbon ion-based RT of sacral chordomas were shown to be a considerable radiogenic late effect, affecting about half of the treated patients. However, only one third of these fractures were clinically symptomatic requiring regular medical care and pain therapy.

Further hazard factor analysis in the future with larger patient numbers will possibly enable the identification of high-risk patients for developing SIFs with the ultimate goal to prevent symptomatic fractures.

## Background

Chordoma is a rare, slow-growing, malignant bone tumor that arises from embryonic remnants of notochord rest cells [1]. These tumors typically manifest in the midline of the neuroaxis with the sacrum being the most common localization. Surgical en-bloc resection is still regarded as the standard of care for sacral chordomas improving local control and disease-free-survival [2].

However, sacral chordomas often reach an enormous size at the time of diagnosis, and the primary surgical treatment bears the risk of substantial postoperative morbidity such as bladder and rectal paralysis, chronic neuropathic pain and/or sensomotoric deficits due to close proximity of the tumor to neurologic structures, which are often infiltrated or encased at the time of diagnosis [3, 4]. Thus, resection margins are very often marginal or positive [2, 5] which is the reason why adjuvant radiotherapy (RT) is commonly used to reduce the risk of local recurrence [6].

Furthermore, definitive high-dose RT is an alternative effective treatment option for patients who are considered inoperable or who refuse surgical treatment. In the last decade, particle therapy (protons/carbon ions) has been established as a preferred radiation technique for sacral chordomas. Several studies reported high local tumor control rates of up to 94% after 5 years when surgery was combined with adjuvant RT [7]. High-dose RT alone has also shown very promising local control rates of up to 88% after 5 years and, in addition, a better preservation of the urinary-anorectal function than surgery [8–11].

However, RT can also cause significant toxicity such as chronic pain syndromes, aggravation of pre-existent motor and sensory functions or even new nerve injuries. In this context, sacral insufficiency fractures (SIFs) may be an important causative factor for increasing morbidity of patients. Several studies already adressed the incidence of SIFs after RT from visceral tumors in the pelvis and found only a low fracture rate ranging from 3 to 11% [12–17]. For sacral chordoma, only one recently published study from the Massachusetts General Hospital (MGH) in Boston addressed concerns regarding SIFs after high-dose radiation treatment; Osler et al. reported a relatively high rate of SIFs following high-dose proton-based RT (47%), in particular when surgery was combined with perioperative radiation treatment [18].

The purpose of our study was to determine the incidence of SIFs after definitive high-dose radiation treatment of sacral chordoma using carbon ion therapy alone or in combination with photons. Furthermore, the time until fracture, the clinical relevance and prognostic factors for development of SIFs were assessed.

## Patients and methods

### Patients

Fifty-six patients with histologically confirmed sacral chordomas were included in this retrospective study. All study patients received high-dose RT at our department in the time period between November 2009 and December 2012. The characteristics of all study patients are summarized in Table 1. The independent ethics committee of the Heidelberg University Medical Faculty approved this retrospective analysis.

**Table 1:** Patients’ characteristics

Characteristics	Value	Percent
Age (y)		
Median	61	
Range	34–84	
Gender (n)		
Female	19	33.9
Male	37	66.1
Most cranial level of the tumor (n)		
L4/5	7	12.5
S1	9	16.1
S2	18	32.1
S3	12	21.4
S4	4	7.1
S5	4	7.1
Os coccygeum	2	3.6
Sacral BMD in planning CT (HU)		
Median	- 18.5	
Range	- 89 – 151	
SIFs (n)	29	51.8
Time to SIF (months)		
Median	11.5	
Range	0–62	
Clinical relevance of SIFs (n)		
Pain	10	34.5
Neurologic deficits	1	3.4

Abbreviations: *BMD* Bone marrow density; *SIFs* Sacral insufficiency fractures; *HU* Hounsfield Units; *CT* Computed tomography; *n* Number; *y* Years

### Treatment

Patients were treated either by carbon ion therapy alone or by a combination of photons and carbon ions based on a CT scan and an additonal MRI scan. Primary RT was applied to 21 patients (37%), whereas the majority of the study population (*n* = 35; 63%) received RT in the postoperative situation (see Table 2). For contouring of the treatment volumes and organs at risk we used the Siemens Oncologist software tools (Siemens, Erlangen, Germany). Macroscopic tumor volume based on MRI was defined as the gross tumor volume (GTV). In most patients (*n* = 49; 88%), we applied a boost plan, in which the clinical target volume (CTV1) covered the GTV and/or the tumor bed plus an additional safety margin of 3–5 mm. For the primary plan, the clinical target volume (CTV2) encompassed not only the CTV1 with safety margins but also the typical ways of tumor spread, i.e. the whole sacrum was commonly included. Furthermore, a small safety margin of 3–7 mm was added to the clinical target volumes (CTV1 and CTV2) to generate the planning target volumes (PTV1 and PTV2). In the subgroup of patients treated with carbon ions alone, the total applied dose was 60–66 Gy RBE applied in 20–22 fractions with a single dose of 3 Gy RBE (see Table 2). In the subgroup of patients who received a bimodal radiation, the treatment concept consisted of an irradiation with 50 Gy photons in 25 fractions (single dose 2 Gy) and 15–24 Gy RBE carbon ions in 5–8 fractions (single dose 3 Gy RBE).

**Table 2:** Treatment

Characteristics		Value	Percent
Resection status (*n*)			
Biopsy		24	42.9
R2		19	33.9
R0/1		13	23.2
Treatment (*n*)			
Primary		42	75.0
S.p. biopsy		21	50.0
S.p. R0/1 resection		10	23.8
S.p. R2 resection		11	26.2
Recurrent		14	25.0
Additional biopsy		1	7.1
Additional R0/1 resection		4	28.6
Additional R2 resection		9	64.3
Radiation dose	ED _2 Gy_		
Carbon ion only:	(α/β = 2)		
60 Gy/3 Gy (RBE)	75.0 Gy	16	28.6
63 Gy/3 Gy (RBE)	78.8 Gy	2	3.6
66 Gy/3 Gy (RBE)	82.5 Gy	15	26.8
IMRT + carbon ion boost			
50 Gy/2 Gy + 15 Gy/3 Gy (RBE)	68.8 Gy	1	1.8
50 Gy/2 Gy + 18 Gy/3 Gy (RBE)	74.5 Gy	1	1.8
50 Gy/2 Gy + 24 Gy/3 Gy (RBE)	80.0 Gy	21	37.5
GTV (*n* = 43; ml)			
Median		244	
Range		5–1188	
CTV1 (*n* = 43; ml)			
Median		522	
Range		64–1743	
CTV2 (*n* = 56; ml)			
Median		937.5	
Range		60–2404	
PTV1 (*n* = 43; ml)			
Median		614	
Range		0–2325	
PTV2 (*n* = 56; ml)			
Median		1067	
Range		84–3099	

Abbreviations: *GTV* Gross tumor volume; *CTV1* Clinical target volume 1 (boost plan); *CTV2* Clinical target volume 2 (primary plan); *PTV1* Planning target volume 1 (boost plan); *PTV2* Planning target volume 2 (primary plan); *RBE* Relative biological effectiveness; *IMRT* Intensity modulated radiotherapy; *n* Number; *ml* Milliliter; *s.p.* Status post

### Follow up and assessment of sacral insufficiency fractures

In the first year after completion of treatment, follow-up examinations including MRI of the pelvis were conducted 6–8 weeks after RT and then at 3-monthly intervals, and in the following years at 6-monthly intervals. The median follow-up was 35.5 months (range 2–83 months). The MR images were reviewed by a board-certified radiologist for presence of SIFs in a blinded manner, i.e. the radiologist did not have informations about the current health status or clinical problems of the study patients. The MR diagnosis of SIFs was based on detection of a fracture line with strongly decreased signal on T1- and T2-weighted images and surrounding medullary edema with decreased signal on T1-weighted images and increased signal on T2-weighted images. If MR imaging patterns indicative for a SIF were found, an additional CT scan of the pelvis was commonly performed to confirm the diagnosis. Medical records were reviewed for correlation of insufficiency fractures with associated clinical symptoms.

### Statistics

Statistical analysis was done using the SAS software version 9.3 (SAS Institute, Cary, NC, USA). A *p*-value of *p* < .05 was considered statistically significant (Chi square, two-sided Wilcoxon test and Log-rank test). Fracture-free survival was plotted according to the Kaplan-Meier method. The log-rank test was used for comparison of the fracture-free survival times between different treatment groups.

Age, tumor volume, sex, radiation dose, bone marrow density, status post operation and status post high sacrectomy were tested for their prognostic significance for predicting SIFs using the Wilcoxon or Chi square test.

## Results

**Fig. 1:**
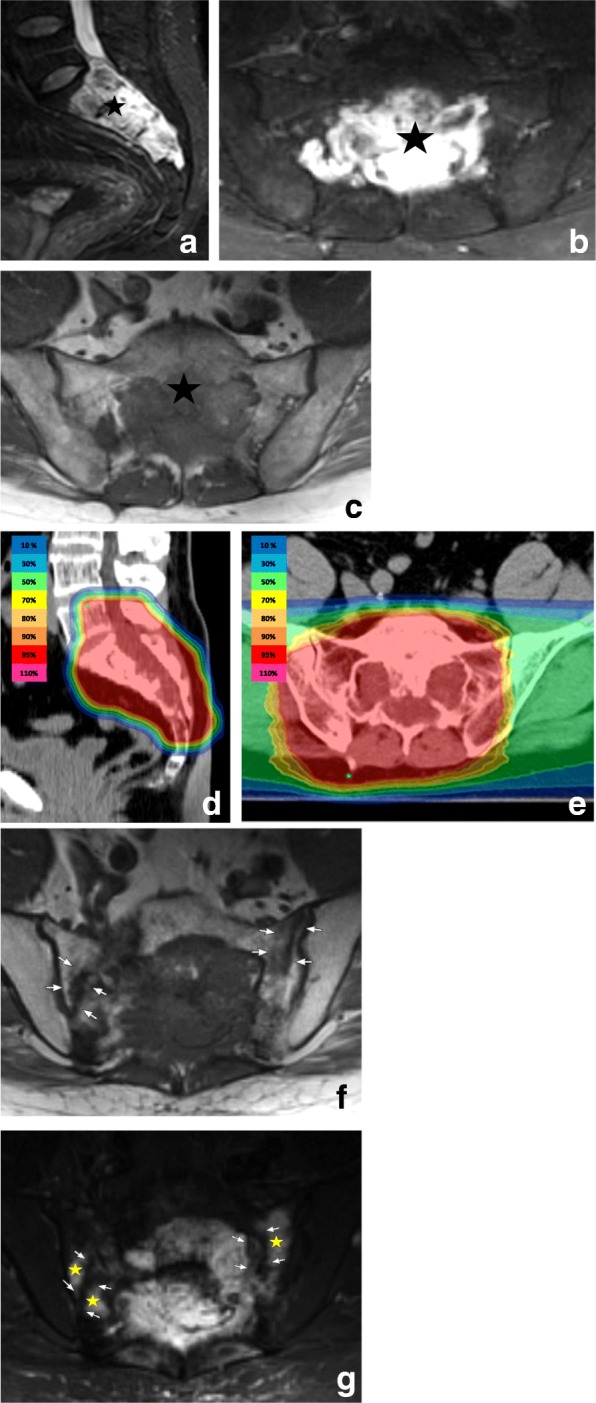
47-year-old man with an extended sacral chordoma (black star) as shown on axial T1- and T2-weighted MR-images before definitive radiotherapy (**a**, **b**, **c**), and on the planning CT with the corresponding dose distribution (**d** and **e**). The post-treatment MRI 12 months after radiation shows sacral insufficiency fractures in both massae laterales of the sacrum (white arrows) with corresponding hypointense linear signal abnormalities on both T1- and T2-weighted MR-images (**f** and **g**). The T2-weighted axial images (**g**) additionally depict the surrounding hyperintense bone marrow edema (yellow stars) which mimics tumor progression

In 52% of the patients (29 of 56 patients), we found SIFs in the follow-up examinations (see Fig. 1). The median time until fracture was 11 months (range 1–62 months). The majority of the affected patients developed the fractures within 2 years after RT (79%; 23 of 29 patients).

**Fig. 2:**
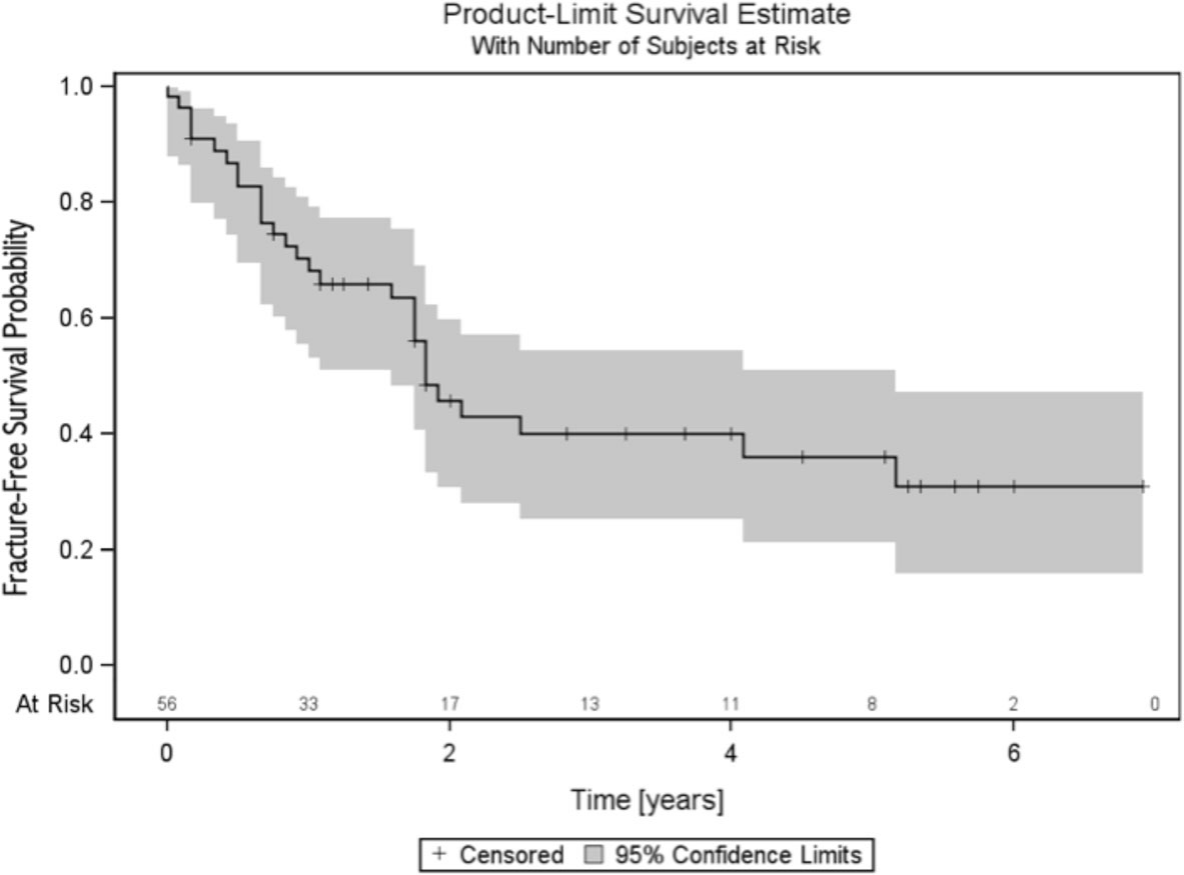
Fracture-free survival probability for the entire patient cohort

**Fig. 3:**
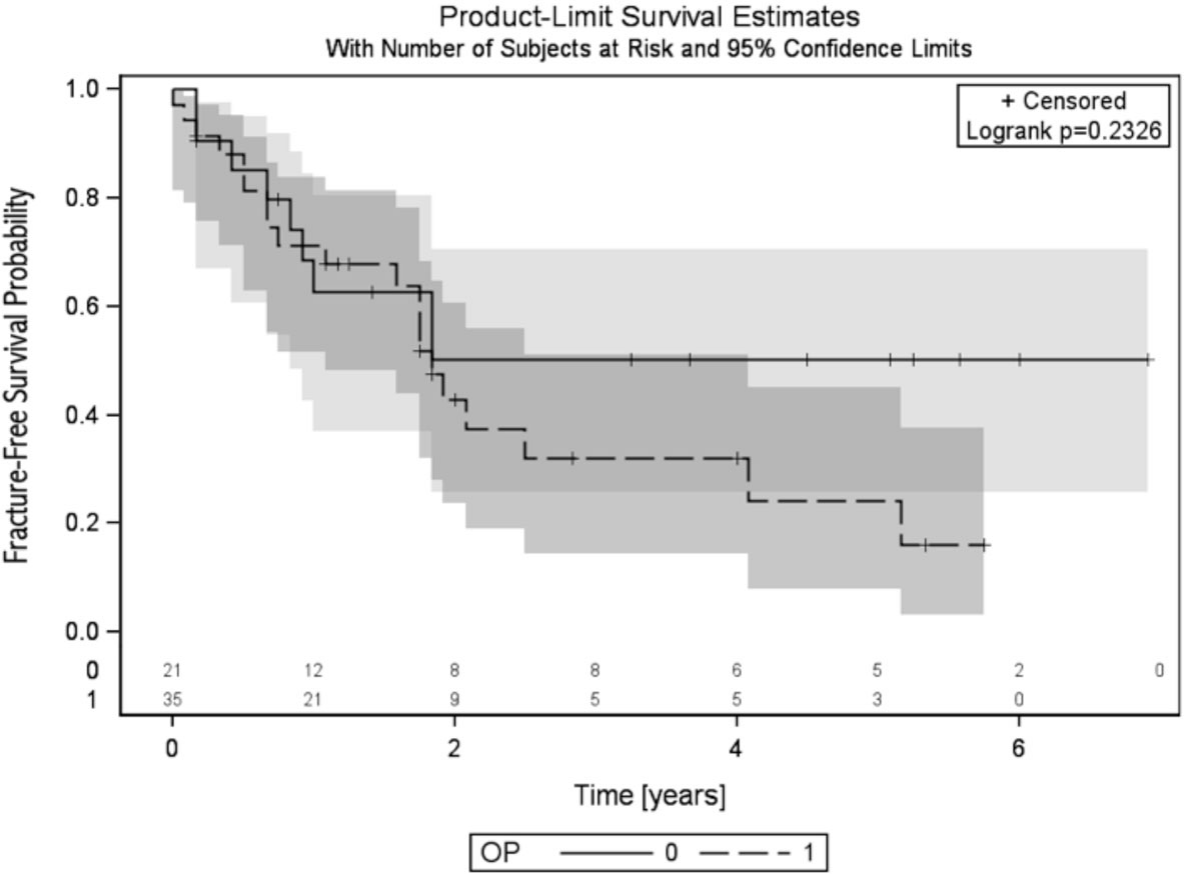
Comparison of the fracture-free survival probabilities between the non-surgical group (solid line) and surgical group (dashed line)

For the overall study population, fracture-free survival probability amounted to values of 0.68 (95% CI, 0.53–0.79) after 1 year, 0.46 (95% CI, 0.31–0.60) after 2 years, and 0.31 (95% CI, 0.16–0.47) after 5 years (see Fig. 2). No statistically significant difference was evident concerning the sacral fracture rates between patients who received a combination therapy of surgery and radiation (57%; 20 of 35) and patients treated with RT alone (43%; 9 of 21) (*p* = 0.23) (see Fig. 3).

In the subgroup of patients treated with sacrectomies and additional RT, the 1-, 2- and 5-year probabilities for freedom of sacral fractures were 0.68 (95% CI, 0.48–0.81), 0.43 (95% CI, 0.24–0.61) and 0.16 (95% CI, 0.03–0.38). In the subgroup of patients treated with definitive RT, the 1-, 2- and 5-year fracture-free survival probabilities amounted to values of 0.63 (95% CI, 0.37–0.80), 0.50 (95% CI, 0.26–0.71) and 0.50 (95% CI, 0.26–0.71), respectively.

**Fig. 4:**
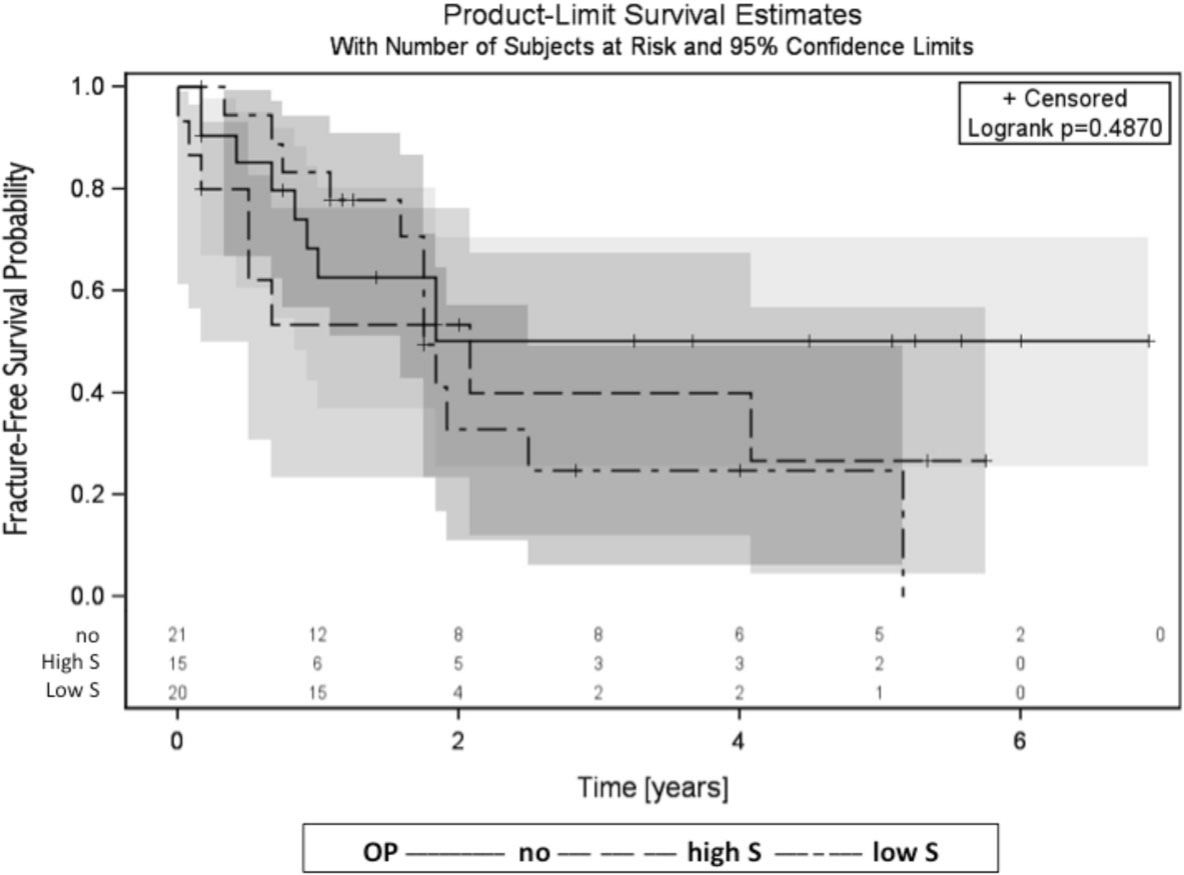
Fracture-free survival probabilities of patients who were only irradiated (solid line) compared with patients who received a high sacrectomy (dashed line; high S in the legend) and patients who received a low sacrectomy (dot-dashed line; low S in the legend)

We also found no statistically significant difference when comparing the high sacrectomy group (i.e., S1-S3 level) with the low sacrectomy group (i.e., below S3 level) and radiation-only group (*p* = 0.49) (see Fig. 4).

In addition, patients’ age and gender, the volume of the tumors, the bone marrow densities prior to RT, the radiation dose and the extent of surgical resection (i.e., high sacrectomy vs. low sacrectomy) were analysed regarding the fracture rates. None of these factors was shown to be statistically significant for prediction of SIFs (see Table 3).

**Table 3:** Results of risk factor analysis related to SIF

Parameter	*p*-value	HR	95% CL
S. p. operation(yes vs. no)	n.s.	0.563	0.189–1.678
S. p. high sacrectomy (yes vs. no)	n.s.	0.919	0.281–3.005
Sacral BMD	n.s.	1.009	0.998–1.021
Female gender (yes vs. no)	n.s.	0.496	0.159–1.542
Age	n.s.	1.016	0.970–1.064
Radiation dose	n.s.	1.002	0.894–1.123
GTV	n.s.	1.0	0.998–1.001

Abbreviations: *HR* Hazard ratio; *CL* Confidence limits; *S.p.* Status post; *BMD* Bone marrow density; *vs.* Versus; *GTV* Gross tumor volume

About one third of the patients with SIFs (34%; 10 of 29 patients) had associated clinical symptoms, of which pain was the main physical impairment (see Table 1). All patients with symptomatic fractures required strong analgesics and often intensive care by a pain therapist; in 1 patient with a SIF, screw fixation of the sacrum was performed.

## Discussion

To date, most toxicity analyses after high-dose radiation of sacral chordoma mostly focused on clinical symptoms (e.g., chronic pain or skin problems) or neurologic impairment (e.g. bladder and rectal paralysis, sensomotoric deficits) [19], but the incidence of SIFs was rarely directly addressed and has therefore probably been underestimated in past studies [2, 5]. Thus, Osler et al. systematically assessed SIFs after high-dose RT of sacral chordoma in a retrospective study and reported about relative high fracture rates in nearly half of the study population, and the proportion was even higher in patients who received a high sacrectomy prior to radiation [18]. In this study, RT was proton-based and carried out in combination with surgical resection or alone in situations where sacral chordomas were not resectable or patients refused their consent for surgery.

In summary, the available study data on SIFs after RT of sacral chordoma are very limited and no data exist concerning the incidence of fractures after carbon ion RT. We therefore aimed to specifically evaluate the rates of SIFs after high-dose carbon- ion-based RT and to compare it with recently published data from the MGH Boston.

In line with the results of Osler et al. [18], we found a relatively high fracture rate of 52% after a median follow-up of about 3 years. The majority of the fractures developed in a time frame of 2 years. However, in 22% of the affected patients fractures were diagnosed later than 2 years after radiation. The slightly higher fracture rate in our study compared to the analysis of the MGH Boston is therefore most likely explainable by the longer median follow-up time in our study (35.5 months vs. 22 months) [18].

An important finding in our study was that about two thirds of SIFs were clinically asymptomatic. Among the symptomatic fractures, new or increased low back pain was by far the leading impairment of affected patients, resulting in mobility restrictions and requiring pain medication.

In contrast to the study data from Osler et al. [18], we did not observe a statistically significant difference of fracture rates between patients treated with a combination of surgery and high-dose radiation compared to high-dose radiation alone. In addition, we analysed patients’ age and gender, the volume of the tumors, the bone marrow density prior to RT, the radiation dose and the extent of surgical resection (i.e., high sacrectomy vs. low sacrectomy) and found none of these factors to be statistically relevant for prediction of SIFs.

Our study has several limitations, among them the retrospective character of the dataset. Secondly, 9 of the 56 study patients were lost to follow-up by 1 year (16%); thus, the fracture rate may even be higher than reported in our study. Moreover, we cannot rule out a small bias, as the 1-year lost to follow-up rate was 19% in the operation group (6 of 32 patients) and 13% in the radiation only group (3 of 24 patients). As a consequence, SIFs might have been more often underdiagnosed in the surgery group than in the radiation-only group, which would have affected our comparison analysis among treatment groups. Another important limitation is the limited number of study patients, particularly with regard to the risk factor analysis.

Additionally, we were not able to statistically analyze the exact further therapies of SIFs, their response to treatment, of the resulting restrictions in patients’ daily living activities and fracture healing in the further course; this study does therefore not provide a reliable response to the question which therapeutic measures need to be taken in the case of symptomatic SIFs for an optimal patient care. Nevertheless, the result of radical resection in patients with sacral chordoma in S2 and higher (60% of patient in our study) would have been a complete urinary and bowel incontinence in at least 80% of the patients [3, 20]. Furthermore chronic neuropathic pain, wound complications and walking difficulties are common side effects of sacrectomy [4, 21]. Therefore, the rate of 18% patients with symptomatic SIFs after irradiation should be assessed against this background.

## Conclusion

About half of patients undergoing high-dose carbon-ion based radiation of sacral chordomas developed sacral fractures during the further course of their disease, mostly within two years after radiation. However, only one third of those fractures resulted in clinical symptoms such as pain or neurologic deficits.

The results of this study emphasize the importance of a regular and close follow-up of patients, whereby radiologists have a pivotal role in the patient care, as the correct interpretation of the imaging scans is a prerequisite for initiation of appropriate therapeutic measures. Further hazard factor analysis in the future will possibly enable the identification of high-risk patients for developing SIFs with the ultimate goal to prevent those patients from symptomatic fractures.

## Abbreviations

CT: Computed tomography
CTV: Clinical target volume
GTV: Gross tumor volume
MGH: Massachusetts General Hospital
MRI: Magnetic resonance tomography
PTV: Planning target volume
RBE: Relative biological effectiveness
RT: Radiotherapy
SIF (s): Sacral insufficiency fracture (s)
